# N3ICD with the transmembrane domain can effectively inhibit EMT by correcting the position of tight/adherens junctions

**DOI:** 10.1080/19336918.2019.1619958

**Published:** 2019-05-27

**Authors:** Junyu Tan, Xixun Zhang, Wenjun Xiao, Xiong Liu, Chun Li, Yuxian Guo, Wei Xiong, Yaochen Li

**Affiliations:** a The central laboratory, Cancer Hospital of Shantou University Medical College, Shantou, China; b Department of Pathology, Cancer Hospital of Shantou University Medical College, Shantou, China

**Keywords:** Breast cancer, Notch3, cell-cell junctions, EMT (epithelial to mesenchymal transition), cell polarity

## Abstract

EMT allows a polarized epithelium to lose epithelial integrity and acquire mesenchymal characteristics. Previously, we found that overexpression of the intracellular domain of Notch3 (N3ICD) can inhibit EMT in breast cancer cells. In this study, we aimed to elucidate the influence of N3ICD or N3ICD combined with the transmembrane domain (TD+N3ICD) on the expression and distribution of TJs/AJs and polar molecules. We found that although N3ICD can upregulate the expression levels of the above-mentioned molecules, TD+N3ICD can inhibit EMT more effectively than N3ICD alone. TD+N3ICD overexpression upregulated the expression of endogenous full-length Notch3 and contributed to correcting the position of TJs/AJs molecules and better acinar structures formation. Co-immunoprecipitation results showed that the upregulated endogenous full-length Notch3 could physically interact with E-ca in MDA-MB-231/pCMV-(TD+N3ICD) cells. Collectively, our data indicate that overexpression of TD+N3ICD can effectively inhibit EMT, resulting in better positioning of TJs/AJs molecules and cell-cell adhesion in breast cancer cells.

**Abbreviations**: EMT: Epithelial-mesenchymal transition; TJs: Tight junctions; AJs: Adherens junctions; aPKC: Atypical protein kinase C; Crb: Crumbs; Lgl: Lethal (2) giant larvae; LLGL2: lethal giant larvae homolog 2; PAR: Partitioning defective; PATJ: Pals1-associated TJ protein

## Introduction

Breast cancer is one of the leading causes of cancer-related deaths in women worldwide, with a crude incidence rate of 1.7 million cases and 521,900 deaths in 2012 [1]. The number of younger women diagnosed with breast cancer has increased in recent years [2]. About 20% to 40% of breast cancer patients eventually develop metastases in distant organs, and nearly all breast cancer-related deaths are caused by metastases rather than the primary tumor, especially those with triple-negative breast cancer (TNBC). TNBC has a more aggressive biological behavior, with worse clinical outcomes, including short durations of relapse-free survival (RFS) and overall survival (OS) compared with non-TNBC [3,4].

In pathological contexts, e.g., the premalignant lesions, on extracellular guidance cues, the epithelial cells defined as surface barrier cells therefore undergo profound morphogenetic changes, collectively referred to as epithelial-mesenchymal transition (EMT) [5], characterized by a transition from an epithelial cobblestone phenotype to an elongated fibroblastic phenotype (mesenchymal phenotype) leading to increased motility and invasion. Recently, although the view that EMT is a key mechanism to trigger cancer metastasis has been challenged, it is becoming increasingly clear that EMT indeed endows epithelium with increased motility and invasiveness, stemness [6], chemoresistance and radioresistance capacities [7–9]. Therefore, EMT is a critical biological process related to the dynamic plastic phenotype of cells during embryonic development and carcinoma progression [10,11].

At the molecular level, epithelial cells show distinct apical versus basolateral polarity established by tight junctions (TJs), adherens junctions (AJs), desmosomes, and so on. They are located at the most apical part of the complex [12] and are important structures for cell-cell adhesion observed in a variety of cell types [13]. The formation and disappearance of TJs or AJs are closely correlated with aggregating and separating cells. TJs play a positive role in an adhesive manner and can prevent cell dissociation [14]. AJs are elements of the cell-cell junction in which cadherin receptors bridge neighboring plasma membranes via their homophilic interactions, and AJs are associated with condensed actin filaments [13,15]. TJs and AJs are important in the formation and maintenance of epithelial integrity and polarity. TJs are therefore the first barrier that epithelial cells must overcome to acquire a mesenchymal phenotype and metastasize [16]. During the acquisition of EMT characteristics, cells lose epithelial cell-cell junctions, and some proteins that promote cell-cell contact such as E-cadherin, E-catenin, and γ-catenin is downregulated and relocates from the cell membrane to the nucleus [11,17]. By contrary, mesenchymal markers, such as vimentin, gain higher expression [17,18].

Notch proteins (Notch1-4) are transmembrane receptors that directly interact with membrane-spanning ligands of neighboring cells (five ligands, including Jagged-1, Jagged-2, Delta-1, Delta-3, and Delta-4) to activate this evolutionarily conserved signaling pathway. Classically, after Notch receptors are triggered by the binding of Notch ligands, the Notch intracellular domain (NICD) is cleaved by a proteinase complex containing γ-secretase. NICD is released from the plasma membrane and translocates into the nucleus, where it forms a complex with RBP-Jk/CBF1, Su(H), Lag-2 (RBP-Jk/CSL), and mastermind-like (MAML) [19,20]. This protein complex will recruit other transcription co-activators and transactivate the transcription of target genes. Activated Notch signaling pathways regulate development by controlling specification of cell fate, cell proliferation, differentiation, and apoptosis during embryonic and postnatal stages, even in tumorigenesis [21]. Over the past few years, it has been shown that the function of Notch signaling in tumorigenesis could be either oncogenic or antiproliferative, and function could be context dependent [22–24]. For example, Notch signaling is associated with many human cancers [25,26] and Notch receptors and ligands have been found to be prognostic markers in human cancers [27–29]. In contrast, Notch signaling has been shown to possess antiproliferative activity rather than oncogenic effect with regard to skin cancer, hepatocellular carcinoma and small cell lung cancer [23]. Our group has focused on the effect of Notch family molecules on the proliferation, EMT, invasion and metastasis of breast cancer cells, and we have found that Notch3 can be a tumor suppressor that inhibits EMT in MDA-MB-231 cells [30–32]. Unfortunately, the functions of Notch3 in the processes of EMT are largely unknown.

Given that the appropriate positioning of TJs/AJs and other related molecules are essential to maintaining intact epithelium, we therefore speculated that the inhibitory effect of N3ICD on EMT may affect the expression and location of TJs/AJs.

In this study, we demonstrated that either ectopic overexpression of intracellular domains (N3ICD) or combined transmembrane and intracellular domains of Notch3 (TD+N3ICD) in MDA-MB-231 cells inhibited EMT by raising the expression levels of TJs/AJs molecules. However, combined overexpression was better at promoting TJs/AJs assembly formations by providing TJs/AJs molecules with proper spatial distribution. These data presented here show a novel role for Notch3, inhibiting EMT by improving the expression of TJs/AJs molecules and restoring their subcellular localization.

### Results

#### Expression of Notch3 was reduced in breast cancer cell lines with relatively high malignancy, closely associated with EMT

**Figure 1.**
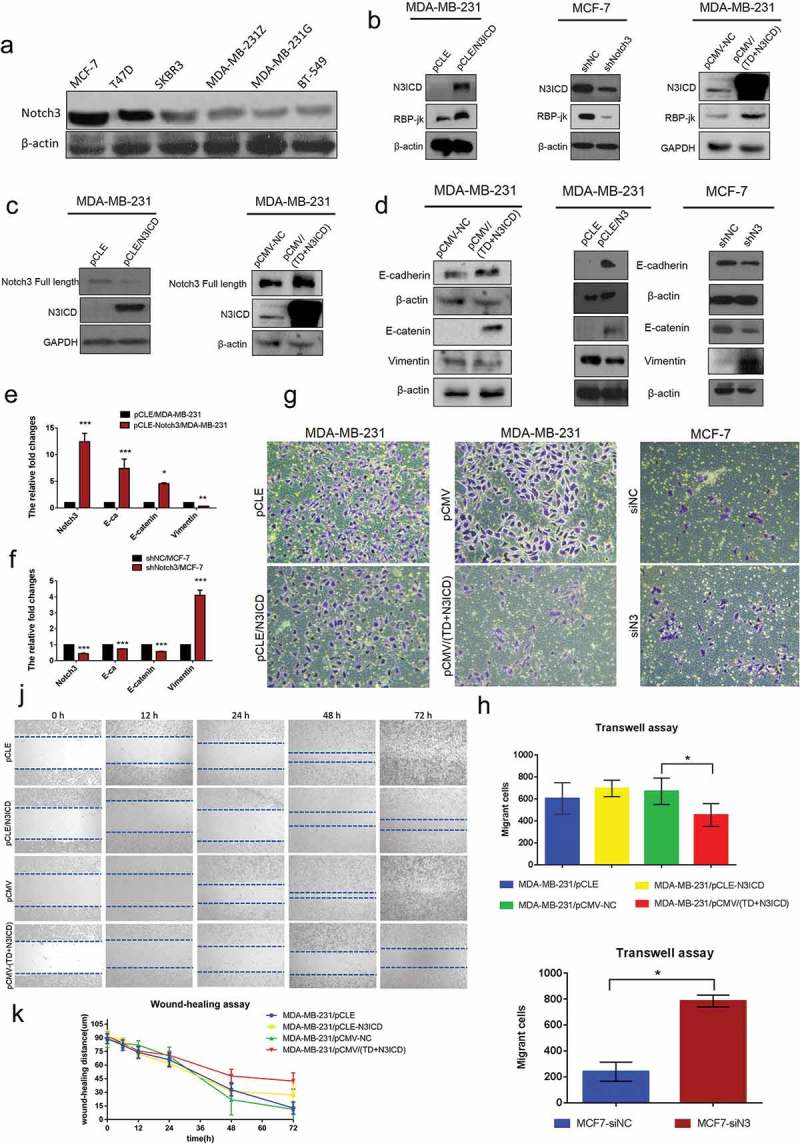
Notch3 overexpression inhibits EMT by the classical Notch signaling pathway in breast cancer cells. (a) Protein expression levels of Notch3 in various human breast cell lines, including MCF-7, T47D, SKBR3, MDA-MB-231Z, MDA-MB-231G, and BT-549, analyzed using western blotting. (b) Effects of Notch3 knockdown or overexpression on expression levels of RBP-jk in MCF-7 or MDA-MB-231 breast cells, respectively, by using western blot analysis. (c) Effects of Notch3 overexpression on expression levels of endogenous full-length Notch3 in MDA-MB-231 breast cells, respectively, by using western blot analysis. (d) Effects of Notch3 knockdown or overexpression on expression levels of endogenous E-ca, E-catenin, and vimentin in MCF-7 or MDA-MB-231 breast cells, respectively, by using western blot analysis. (e) Effects of Notch3 overexpression on expression levels of endogenous E-ca, E-catenin, and vimentin in MDA-MB-231 breast cells by using qRT-PCR. (f) Effects of Notch3 knockdown on expression levels of endogenous E-ca, E-catenin and vimentin in MCF-7 breast cells by using qRT-PCR. (g) MCF-7 or MDA-MB-231 cells were subjected to the transwell assay under knockdown or overexpression of Notch3, respectively, including N3ICD or TD+N3ICD. Cells migrating across the membrane were imaged with a bright-field microscope at ×40 magnification. (h) Quantitative data of Matrigel non-coated transwell assays. MDA-MB-231 cells were transfected with pCLE, pCLE-N3ICD, pCMV or pCMV-(TD+N3ICD). Migrant cells were counted in five random fields. (i) Quantitative data of Matrigel non-coated transwell assays. MCF-7 cells were transfected with siNC or siN3. Migrant cells were counted in whole fields. (j) Wound healing assays were performed with MDA-MB-231 cells with ectopic overexpression of N3ICD or TD+N3ICD. Representative images. (k) Quantitative wound recovery data after 0, 6, 12, 24, 48 and 72 h in cell culture. Data are presented as the mean ± SD of three independent experiments, and asterisks indicate statistical significance (*, p < 0.05; **, p < 0.01; ***, p < 0.001).

We first compared the relative endogenous Notch3 expression levels by western blotting in a panel of breast cancer cell lines previously classified as four subtypes, namely MCF-7 and T47D (ERα/PgR-positive luminal mammary carcinoma cells), SKBR3 (Her2 (Neu/ErbB-2)-overexpressing human breast cancer cell line), MDA-MB-231 (G and Z indicating two strains stored in different laboratories) and BT549 (triple-negative/basal-like breast cancer cells, TNBC). According to western blot analysis, Notch3 showed lower expression levels in those tumor cell lines with relatively high malignancy, such as MDA-MB-231 and BT549 TNBC. In contrast, MCF-7 and T47D exhibited higher expression levels of Notch3 (Figure 1(a)).

To investigate the potential role of Notch3 signaling in breast cancer cells, we, respectively, established stable N3ICD- as well as TD+N3ICD-overexpressing transfectants in low Notch3-expressing MDA-MB-231 cells by transfecting pCLE/N3ICD or pCMV-(TD+N3ICD) plasmids. In addition, we also generated stable Notch3 knockdown cells in high Notch3-expressing MCF-7 cells by transfecting pGPU6/GFP/Neo/shN3.

To assess whether the classical Notch signaling pathway was activated along with N3ICD overexpression, the expression level of RBP-jk was determined by western blotting. The results showed that the expression levels of RBP-jk increased or decreased, accompanied by overexpression of N3ICD or knockdown of Notch3. Likewise, there was also an obvious increase in the expression level of RBP-jk along with the ectopic overexpression of TD+N3ICD (Figure 1(b)). Of note, we observed an important phenomenon that the endogenous full-length Notch3 was upregulated with ectopic overexpression of TD+N3ICD, but it was not affected by overexpression of N3ICD (Figure 1c). We further observed the effect of N3ICD on EMT-associated markers, such as E-cadherin (E-ca), E-catenin, and vimentin by qRT-PCR and western blotting. Western blot analysis showed that the expression levels of E-ca and E-catenin were upregulated, whereas vimentin expression was downregulated with N3ICD or TD+N3ICD overexpression in MDA-MB-231 cells compared with control cells. On the contrary, the downregulated expression levels of E-ca and E-catenin as well as upregulated expression level of vimentin were evident in MCF-7/shRNA-N3 cells compared with MCF-7/shNC cells (Figure 1d). These results were further verified by qRT-PCR analysis (Figure 1(e,f)).

Next, the ability of cell migration was investigated and compared by the transwell migration assay under the prerequisite of overexpressing Notch3, including N3ICD and TD+N3ICD, or knocking down Notch3. The transwell assay revealed that although few pCLE/N3ICD cells migrated through the membrane compared with the control cells (MDA-MB-231 cell transfected with empty vector, pCLE) after 48 h, there was no statistical difference. Nevertheless, there was a significant difference between MDA-MB-231/pCMV-(TD+N3ICD) and control cells in their capacity to migrate through the membrane, where fewer MDA-MB-231/pCMV-(TD+N3ICD) cells passed through the transwell membrane (Figure 1(g,h)). Meanwhile, Notch3 knockdown significantly increased MCF-7 cell migration across the transwell membrane (Figure 1(g,i)), indicating that TD+N3ICD3 was more effective at inhibiting breast cancer cell migration compared with N3ICD. We further assessed and compared the recovery wound healing by expressing N3ICD and TD+N3ICD. The results showed that migration rates were significantly lower after overexpression of N3ICD or TD+N3ICD compared with control cells. Furthermore, wound healing ability of the MDA-MB-231/pCLE-N3ICD cells was better compared with MDA-MB-231/pCMV-(TD+N3ICD) cells (Figure 1(j,k)). Taken together, Notch3 may participate in inhibiting EMT in breast cancer by the classical or non-classical signaling pathway, and the transmembrane domain may play an important role during this process.

#### Tight junctions molecules were upregulated and located in appropriate positions after overexpression of TD+N3ICD

**Figure 2.**
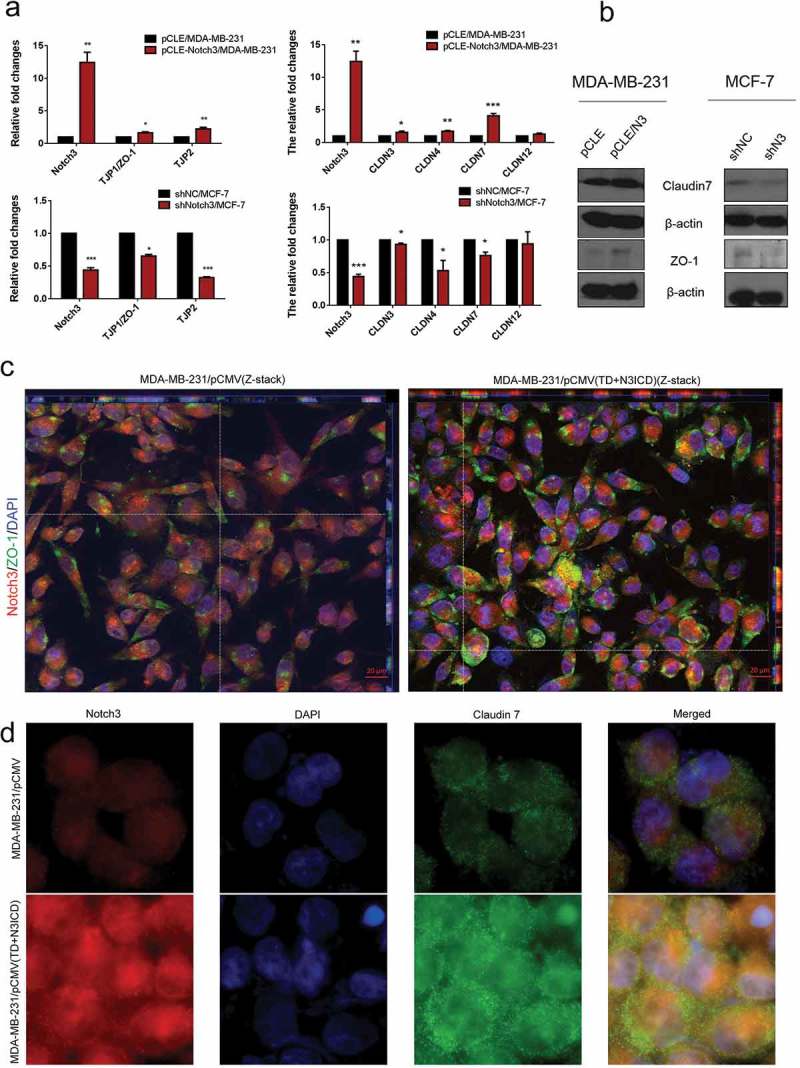
Ectopic overexpression of TD+N3ICD upregulates the expression of tight junctions molecules and promotes their appropriate localization (a) Effects of Notch3 knockdown or overexpression on expression levels of tight junctions molecules in MCF-7 or MDA-MB-231 breast cells, respectively, by using qRT-PCR. (b) Effects of Notch3 knockdown or overexpression on expression levels of tight junctions molecules in MCF-7 or MDA-MB-231 breast cells, respectively, by using western blotting. (c) Double-label immunofluorescence staining of Notch3 (red color) and ZO-1 (green color) and Z-stack analysis in MDA-MB-231/pCMV and MDA-MB-231/pCMV-(TD+ N3ICD) cells, 40 ×. (d) Double-label immunofluorescence staining of Notch3 (red color) and Claudin 7 (green color) in MDA-MB-231/pCMV and MDA-MB-231/pCMV-(TD+ N3ICD) cells, 40 ×. Data are presented as the mean ± SD of three independent experiments, and asterisks indicate statistical significance. (*, p < 0.05; **, p < 0.01; ***, p < 0.001).

**Figure 3.**
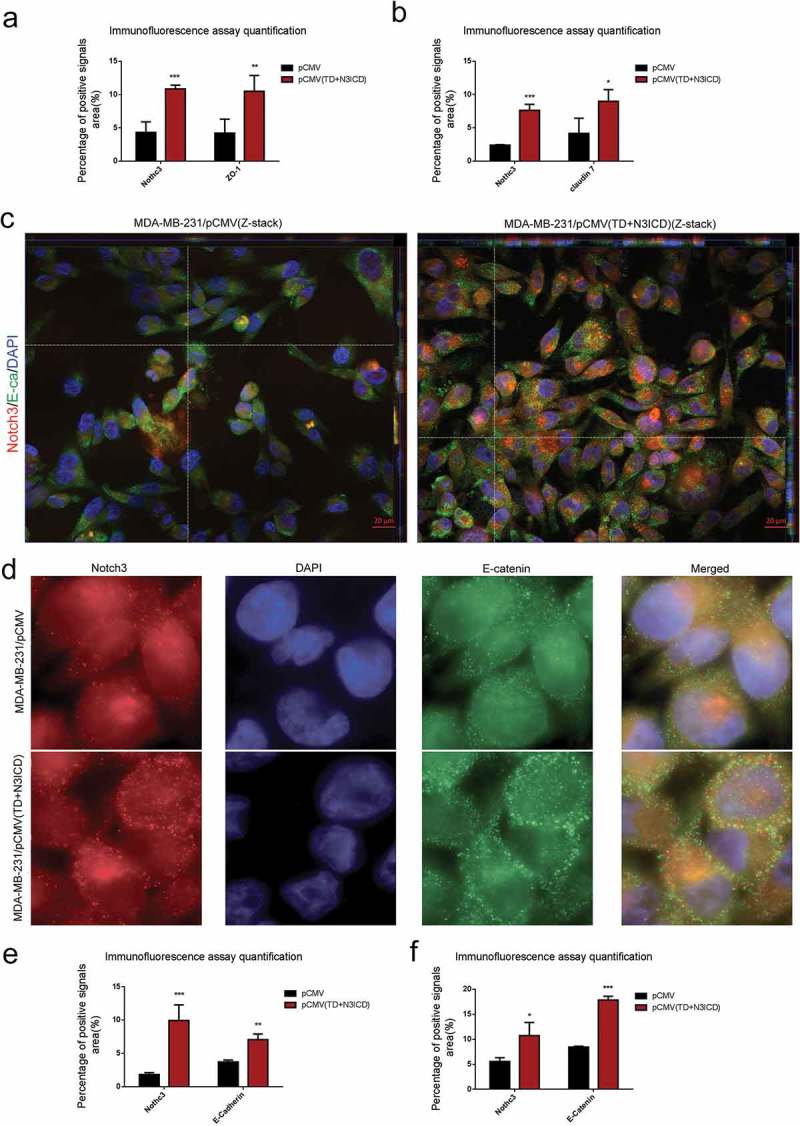
Ectopic overexpression of TD+N3ICD upregulates the expression of adherens junction molecules and promotes their appropriate localization (a) Quantification of positive ZO-1 signals by calculating the positive area combined with the signal intensity. (b) Quantification of positive Claudin 7 signals by calculating the positive area combined with the signal intensity. (c) Double-label immunofluorescence staining of Notch3 (red color) and E-cadherin (green color) and Z-stack analysis in MDA-MB-231/pCMV and MDA-MB-231/pCMV-(TD+ N3ICD) cells, 40 ×. (d) Double-label immunofluorescence staining of Notch3 (red color) and E-catenin (green color) in MDA-MB-231/pCMV and MDA-MB-231/pCMV-(TD+ N3ICD) cells, 100 ×. (e) Quantification of positive E-cadherin signals by calculating the positive area combined with the signal intensity. (f) Quantification of positive E-catenin signals by calculating the positive area combined with the signal intensity. Data are presented as the mean ± SD of three independent experiments, and asterisks indicate statistical significance. (*, p < 0.05; **, p < 0.01; ***, p < 0.001).

To investigate whether overexpression of N3ICD could restore the expression levels and positions of TJs molecules, a panel of experiments was carried out. First, mRNA expression levels of tight junction protein (TJP) family members were examined by qRT-PCR. The results revealed that the mRNA expression levels of TJP1/ZO-1 and TJP2 were upregulated or downregulated, respectively, with overexpression or knockdown of Notch3 (Figure 2(a)). In addition, we also surveyed the expression levels of another tight junction family, the claudin gene (CLDN) family, including CLDN3, CLDN4, CLDN7, and CLDN12. qRT-PCR revealed that the CLDN3, 4 and 7 were expressed at high levels in MDA-MB-231/pCLE-N3ICD compared to MDA-MB-231/pCLE cells. On the contrary, their expression was also downregulated with Notch3 knockdown. Western blot analysis was performed to evaluate the expression levels of ZO-1 and claudin 7. The results showed that there were positive correlations between Notch3 and ZO-1 or claudin 7 (Figure 2(b)). Next, we used double-label immunofluorescence staining and Z-stack analysis to determine whether these TJs molecules possessed correct location, and whether there was TJs formation between neighbor cells. The Z-stack images collected at 1.11 μm sections were presented in Figure S1 A and B. As shown in Figure 2(c), immunofluorescence staining and Z-stack image showed that the intensities of ZO-1 and Notch3 positive signals in MDA-MB-231/pCMV(TD+N3ICD) cells were significantly stronger than that in control cells (Figure 2(c)). Besides, we found that the TD+N3ICD-overexpressing group had good distribution and clear tight conjunction of ZO-1 (Figure 2(c), right), while control cells displayed an irregular distribution (Figure 2(c), left). Similarly, the stronger positive claudin 7 signals showed a uniform distribution closely surrounding the cell membrane in MDA-MB-231/pCMV-(TD+N3ICD) cells (Figure 2(d), lower row), whereas very weak signals were seen in MDA-MB-231/pCMV cells (Figure 2(d), upper row). The quantification of ZO-1 and claudin 7 positive signals were calculated by Image J software, respectively. The quantification results showed that the combined intensities of ZO-1 and claudin 7 positive signals in MDA-MB-231/pCMV-(TD+N3ICD) cells were significantly stronger than that in control cells (Figure 3(a,b)). In summary, these data further demonstrated that ectopic overexpression of TD+N3ICD can upregulate TJs molecules and improve or promote TJs formation in breast cancer cells.

#### Ectopic overexpression of TD+N3ICD upregulated the expression of adherens junctions molecules and promoted their appropriate positioning

Double-label immunofluorescence staining and Z-stack analysis were performed to test whether the upregulated AJs molecules could functionally mediate cell-cell adhesion through homophilic interactions with their neighbors. The Z-stack images collected at 1.11 μm sections were presented in Figure S2 A and B. The results showed that the intensity of positive signals and the amount of E-ca (green color) were stronger and much more in MDA-MB-231/pCMV-(TD+N3ICD) than in control MDA-MB-231/pCMV cells. At the same time, we noticed that E-ca encircled the cell membrane, co-localized with Notch3 (red color) and formed the belt-like adherens structures, encircling the cell membrane, namely the zonula adherens, where parts of them reached the whole cell membrane in MDA-MB-231/pCMV-(TD+N3ICD) cells (Figure 3(c), right). Evident connections formed between neighboring cells. In contrast, in the control of MDA-MB-231/pCMV cells, the distribution of E-ca showed a punctate or focal shape (Figure 3(c), left).

In addition, E-catenin is another important AJs molecule. Double-label immunofluorescence staining also showed a much stronger intensity of positive signals in MDA-MB-231/pCMV-(TD+N3ICD) cells than that in MDA-MB-231/pCMV cells. Similar to the normal epithelial cells, the positive signals were clearly located on the cell membrane in MDA-MB-231/pCMV-(TD+N3ICD) cells (Figure 3(d), lower row), whereas the positive signals in MDA-MB-231/pCMV had moved into the cytoplasm and nucleus (Figure 3(d), upper row). The quantification of E-ca and E-catenin positive signals was calculated by Image J software, respectively. The results showed that the areas combined intensities of E-ca and E-catenin positive signals in MDA-MB-231/pCMV-(TD+N3ICD) cells were significantly more than that in control cells (Figure 3(e,f)). Collectively, these data indicated that TD+N3ICD not only increased expression of adherens molecules, but also promoted their appropriate positioning.

#### Ectopic overexpression of TD+N3ICD upregulated the expression levels of scaffolding proteins and promoted their appropriate positioning

**Figure 4.**
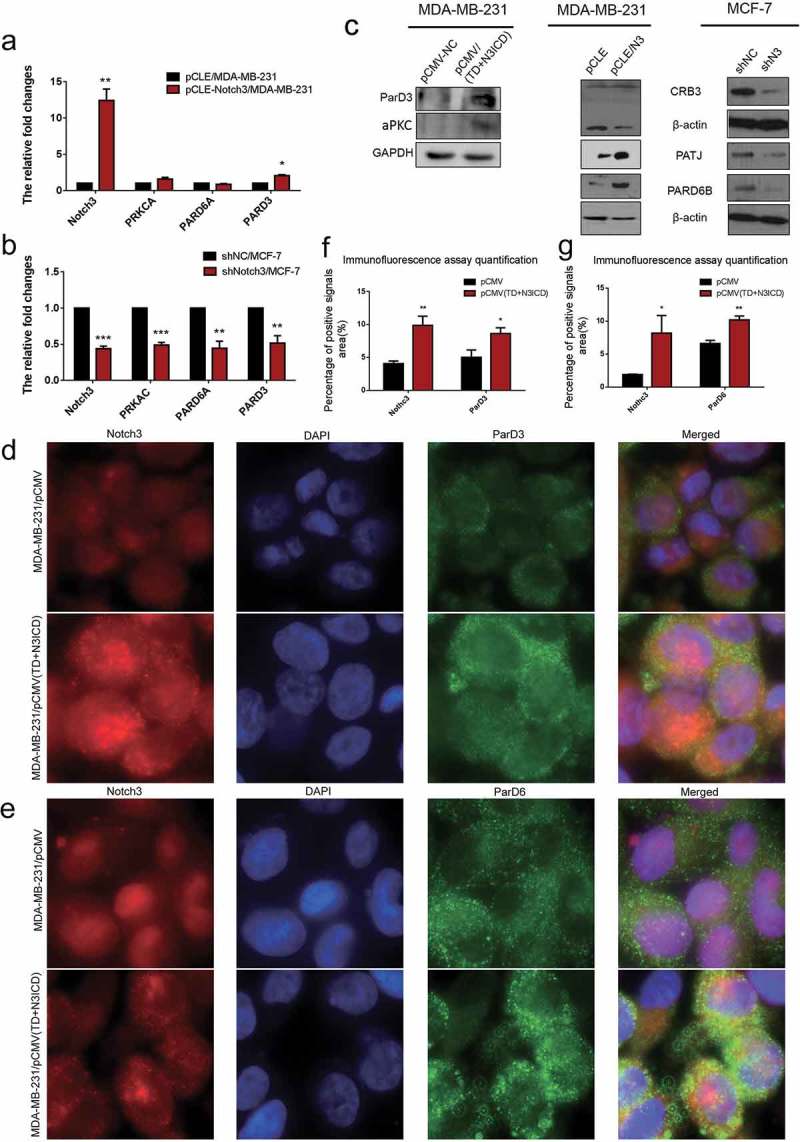
Ectopic overexpression of TD+N3ICD upregulates the expression of PAR-3-aPKC-PAR-6 complex components and promotes their appropriate localization. (a) Effects of Notch3 overexpression on expression levels of PAR-3-aPKC-PAR-6 complex in MDA-MB-231 breast cells by using qRT-PCR. (b) Effects of Notch3 knockdown on expression levels of PAR-3-aPKC-PAR-6 complex in MCF-7 breast cells by using qRT-PCR. (c) Effects of Notch3 knockdown or overexpression on expression levels of PAR-3-aPKC-PAR-6 complex in MCF-7 or MDA-MB-231 breast cells, respectively, by using western blotting. (d) Double-label immunofluorescence staining of Notch3 (red color) and ParD3 (green color) in MDA-MB-231/pCMV and MDA-MB-231/pCMV-(TD+ N3ICD) cells, 40 ×. (e) Double-label immunofluorescence staining of Notch3 (red color) and ParD6 (green color) in MDA-MB-231/pCMV and MDA-MB-231/pCMV-(TD+ N3ICD) cells, 40 ×. (f) Quantification of positive ParD3 signals by calculating the positive area combined with the signal intensity. (g) Quantification of positive ParD6 signals by calculating the positive area combined with the signal intensity. Data are presented as the mean ± SD of three independent experiments, and asterisks indicate statistical significance (*, p < 0.05; **, p < 0.01; ***, p < 0.001).

**Figure 5.**
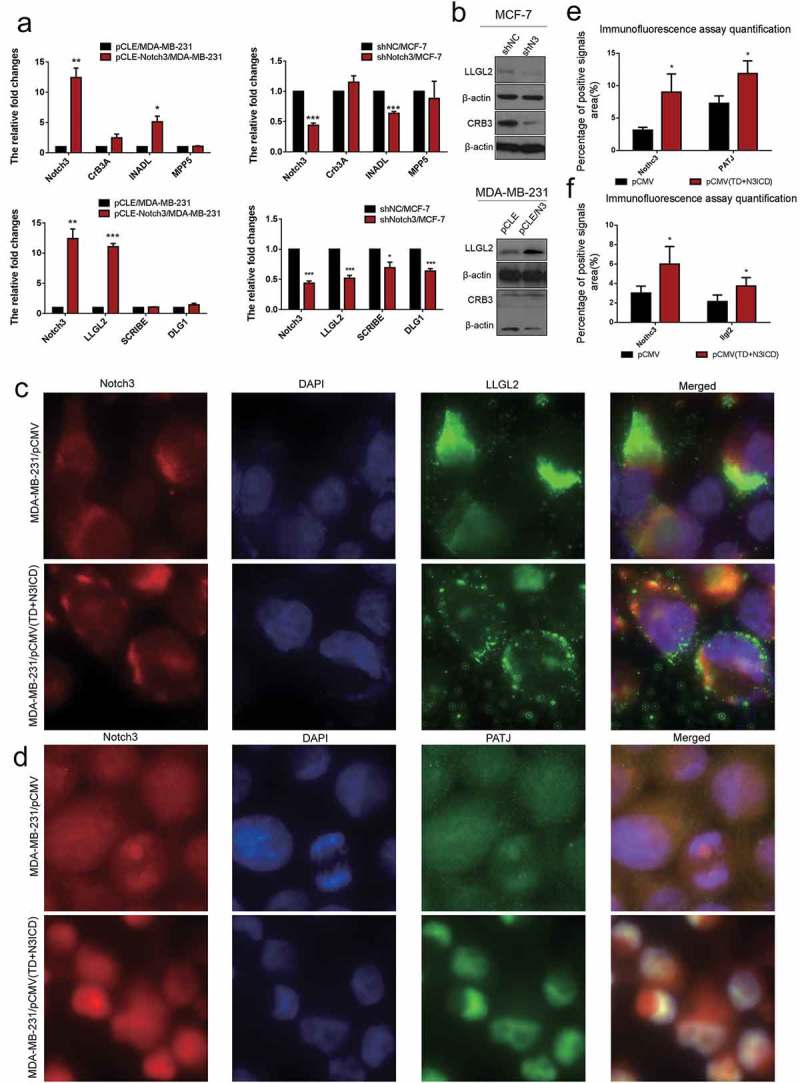
Ectopic overexpression of TD+N3ICD upregulates the expression of cell basal and lateral polarity complex components and promotes their appropriate localization. (a) Effects of Notch3 knockdown or overexpression on expression levels of cell basal and lateral polarity complex components in MCF-7 or MDA-MB-231 breast cells, respectively, by using qRT-PCR. (b) Effects of Notch3 knockdown or overexpression on expression levels of cell basal and lateral polarity complex components in MCF-7 or MDA-MB-231 breast cells, respectively, by using western blotting. (c) Double-label immunofluorescence staining of Notch3 (red color) and LLG2 (green color) in MDA-MB-231/pCMV and MDA-MB-231/pCMV-(TD+ N3ICD) cells, 40 ×. (d) Double-label immunofluorescence staining of Notch3 (red color) and PATJ (green color) in MDA-MB-231/pCMV and MDA-MB-231/pCMV-(TD+ N3ICD) cells, 40 ×. (e) Quantification of positive PATJ signals by calculating the positive area combined with the signal intensity. (f) Quantification of positive LLGL2 signals by calculating the positive area combined with the signal intensity. Data are presented as the mean ± SD of three independent experiments, and asterisks indicate statistical significance (*, p < 0.05; **, p < 0.01; ***, p < 0.001).

The scaffolding proteins are the important components of TJs that are required for normal apicobasal polarity [33]. To determine the effect of N3ICD overexpression on expression and location of scaffolding proteins, the expression of scaffolding molecules such as the catalytic subunit α of protein kinase A (PRKCA), partitioning defective 6 homolog alpha (PARD6A) and partitioning defective 3 homolog (PARD3) were detected. The expression of PARD6A and PARD3 was upregulated or reduced in mRNA levels with N3ICD overexpression or knockdown, and PRKCA showed no significant changes with N3ICD overexpression (Figure 4(a,b)). We also determined their protein expression. Western blot analysis verified that PARD6 and PARD3 were positively regulated by Notch3. In addition, we found that N3ICD overexpression could upregulate the expression of atypical protein kinase C (aPKC), Crumbs protein homolog 3 (CRB3), and PALS1-associated tight junction protein (PATJ) (Figure 4(c)). These observations prompted us to analyze the subcellular localization of PARD3, PARD6, and PATJ. Immunofluorescence staining showed that a larger number of positive signals of PARD3 and PATJ were located on the lateral side of the MDA-MB-231/pCMV-(TD+N3ICD) cells (Figure 4(d), lower row and Figure 5(d), lower row). In contrast, the positive signals were significantly reduced in MDA-MB-231/pCMV cells (Figure 4(d), upper row and Figure 5(d), upper row). For the PARD6 staining, more stronger signals encircling the membrane were observed in MDA-MB-231/pCMV-(TD+N3ICD) cells (Figure 4(e), lower row) when compared with control cells (Figure 4(e), upper row). The quantification of PARD3, PARD6, and PATJ positive signals was calculated by Image J software, respectively. The results showed that the areas combined intensities of PARD3, PARD6, and PATJ positive signals in MDA-MB-231/pCMV-(TD+N3ICD) cells were significantly more than that in control cells (Figures 4(f,g) and 5(e)). Taken together, ectopic expression of TD+N3ICD promoted the recruitment of scaffolding proteins, such as PAR-3-aPKC-PAR-6 complex and Pals1-PATJ complex, to TJs so that cell polarity was regulated.

### Ectopic overexpression of Notch3 inhibited EMT cell polarity

To investigate the effect of N3ICD on cell polarity proteins, the expression levels and subcellular location of the Crumbs/Scribble complex were determined. According to the qRT-PCR results, except for InaD-like protein (INADL/PATJ) and the lethal (2) giant larvae protein homolog 2 (LLGL2), other Crumbs/Scribble molecules were not affected by N3ICD overexpression or Notch3 knockdown. There was an evident positive correlation between Notch3 and INADL or LLGL2 (Figure 5(a)). Western blot analysis revealed that the expression levels of LLGL2 were, respectively, upregulated or downregulated along with overexpressing or knocking down Notch3 (Figure 5(b)). To elucidate the effect of TD+N3ICD on LLGL2 distribution, double-label immunofluorescence staining was employed. The results showed that LLGL2 exhibited a typical asymmetric polar distribution in MDA-MB-231/pCMV cells (Figure 5(c), upper row). However, relatively speaking, LLGL2 evenly localized to multiple locations in the MDA-MB-231/pCMV-(TD+N3ICD) cells (Figure 5(c), lower row). The quantification of LLGL2 positive signals was calculated by Image J software. The results showed that the areas of LLGL2 positive signals in MDA-MB-231/pCMV-(TD+N3ICD) cells were significantly more than that in control cells (Figure 5(f)). Collectively, ectopic overexpression of Notch3 forces alteration of LLGL2 subcellular positions, which may lead to a failure of directional migration of breast cancer cells.

### Ectopic overexpression of TD+N3ICD promoted acinar formation in 3D culture

**Figure 6.**
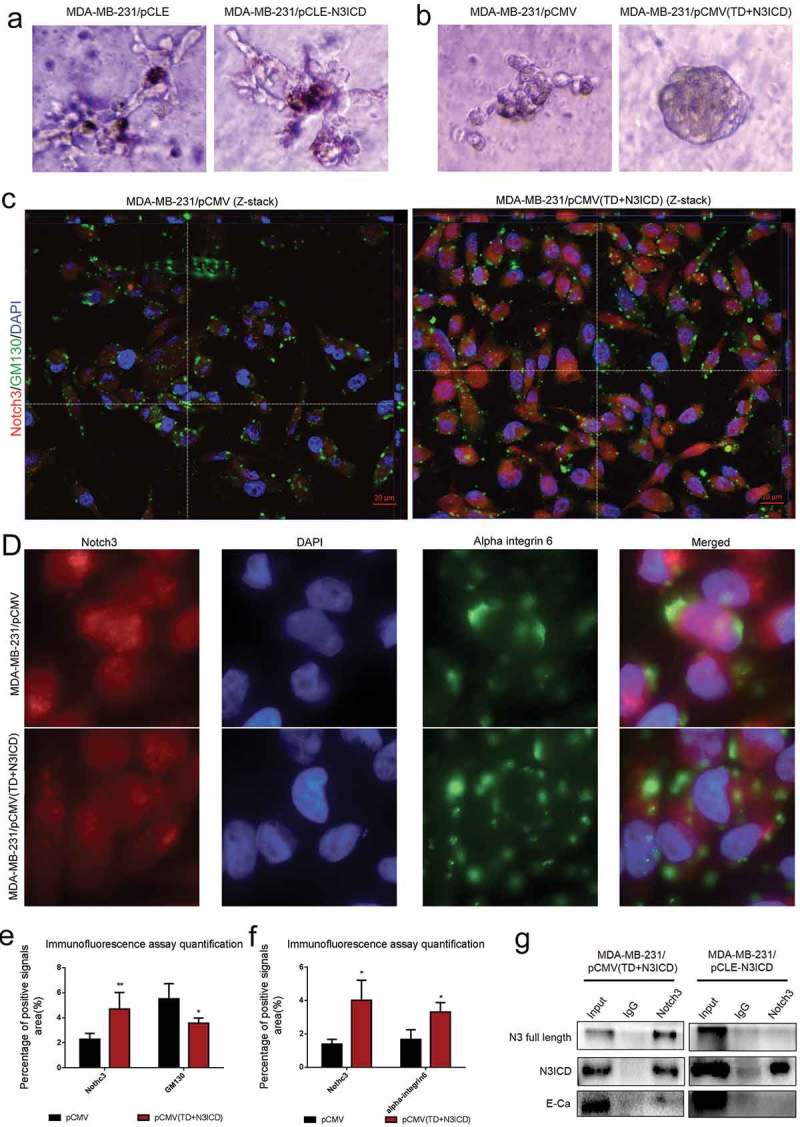
The upregulated endogenous full-length Notch3, which interacts with E-ca, can promote acinar formation and change the distributions of GM130 and alpha-integrin 6 in MDA-MB-231 cells/pCMV-(TD+ N3ICD) cells. (a) Acinar formation assay. The acinar structures of MDA-MB-231 cells transfected with pCLE and pCLE/N3ICD which cultured in Matrigel for 17 days, 10 ×. (b) The acinar structures of MDA-MB-231 cells transfected with pCMV and pCMV/(TD+N3ICD) which cultured in Matrigel for 17 days, 10 ×. (c) Double-label immunofluorescence staining of Notch3 (red color) and GM130 (green color) and Z-stack analysis in MDA-MB-231/pCMV and MDA-MB-231/pCMV-(TD+ N3ICD) cells, 100 ×. (d) Double-label immunofluorescence staining of Notch3 (red color) and alpha-integrin 6 (green color) in MDA-MB-231/pCMV and MDA-MB-231/pCMV-(TD+ N3ICD) cells, 40 ×. (e) Quantification of positive GM130 signals by calculating the positive area combined with the signal intensity. (f) Quantification of positive alpha-integrin 6 signals by calculating the positive area combined with the signal intensity. (g) Co-immunoprecipitation followed by western blotting was performed to detect physical associations between endogenous Notch3 and E-ca. The anti-Notch3 antibody was used to pull down, and the anti-Notch3, E-ca antibodies were used to probe the IP complexes. Input was used as a positive control and IgG was used as a negative control. Data are presented as the mean ± SD of three independent experiments, and asterisks indicate statistical significance (*, p < 0.05; **, p < 0.01; ***, p < 0.001).

It is well known that normal mammary gland epithelial cells have several distinguishing histological features including a polarized morphology, specialized cell-cell contacts, attachment to an underlying basement membrane, and organized acinar structures. We asked whether ectopic overexpression of N3ICD or TD+N3ICD in MDA-MB-231 can generate organized acinar structures. We used Matrigel 3D culture which can simulate approximate reconstruction of the basement membrane. The results showed that the control MDA-MB-231 cells that were transfected with empty vectors, including pCMV or pCLE, began to proliferate quickly, and exhibited several oncogenic phenomena such as, invasive behaviors, disorganized architectural morphology in the Matrigel (Figure 6(a) left and B left). Of note, no obvious differences were observed in the acinar structures and configuration from those MDA-MB-231 cells transfected with pCLE-N3ICD when compared with control cells (Figure 6(a) right). On the contrary, the MDA-MB-231 cells transfected with pCMV-(TD+N3ICD) began to proliferate on reconstituted basement membrane and make well-organized 3D spherical architectures (Figure 6(b) right). In summary, these data demonstrate that the overexpression of N3ICD combined with the transmembrane domain can morphologically promote better 3D acinar structures formation.

### Ectopic overexpression of Notch3 inhibited Golgi compaction

To further confirm that overexpression of N3ICD can inhibit EMT in breast cancer cells, Golgi structural features were determined by double-label immunofluorescence staining with antibodies against the *cis*-Golgi marker GM130. First, we found that GM130 expression level was reduced in MDA-MB-231/pCMV-(TD+N3ICD) cells compared to the control cells. We next observed that the Golgi had different distribution between MDA-MB-231/pCMV control cells and MDA-MB-231/pCMV-(TD+N3ICD) cells with Apotome Z-stack function. The Z-stack images collected at 1.11 μm sections were presented in Figure S3 A and B. The results revealed that the compacted Golgi structures were at the leading edge of the polarized MDA-MB-231/pCMV control cells (Figure 6(c), left). Compared with the control cells, MDA-MB-231/pCMV-(TD+N3ICD) cells had less compacted Golgi, which contained smaller and fewer Golgi elements (Figure 6(c), right). The quantification of GM130 and alpha-integrin 6 positive signals was calculated by Image J software, respectively. The results showed that the areas combined intensities of GM130 positive signals in MDA-MB-231/pCMV-(TD+N3ICD) cells were significantly less than that in control cells (Figure 6(e)). These data suggest that ectopic overexpression of Notch3 can suppress EMT by inhibiting Golgi compaction. We also determined the expression and distribution of alpha-integrin 6 by double-label immunofluorescence staining. The results showed that there was a slight increase in expression level (Figure 6(f)), but that its distribution became more scattered on the cell membrane of MDA-MB-231/pCMV-(TD+N3ICD) cells (Figure 6(d), lower row) compared with the control MDA-MB-231/pCMV cells (Figure 6(d), upper row). Taken together, these data demonstrate that the overexpression of N3ICD combined with the transmembrane domain can change the subcellular distribution of some EMT-related molecules.

### The upregulated endogenous full-length Notch3 is a key transmembrane molecule to redistribute TJs/AJs back to the right position in MDA-MB-231/pCMV-(TD+N3ICD) cells

We further asked whether the full-length Notch3 could be part of a supramolecular complex in MDA-MB-231 cells transfected with pCMV-(TD+N3ICD) as indicated by their colocalization on the cell surface verified by double-label immunofluorescence staining. To answer this question, we performed Co-immunoprecipitation (Co-IP) assays with specific anti-Notch3 antibody. Co-IP revealed that Notch3 could pull down endogenous E-ca and full length Notch3 in MDA-MB-231/pCMV-(TD+N3ICD) cells. However, no obvious proteins were pulled down in MDA-MB-231/pCLE-N3ICD cells beside N3ICD (Figure 6(g)). These data suggest that the full-length Notch3 and E-cadherin may physically interact in MDA-MB-231/pCMV-(TD+N3ICD) cells.

## Discussion:

Based on our previous finding that Notch3 overexpression can inhibit EMT in MDA-MB-231 [30], in this study, we overexpressed N3ICD and N3ICD combined with the transmembrane domain (TD+N3ICD) respectively, and compared their EMT inhibiting efficiency. We found that both the overexpression of N3ICD and TD+N3ICD can upregulate the expression levels of the TJs/AJs, scaffolding proteins and polar molecules, but only overexpression of TD+N3ICD can promote better 3D acinar structures formation and inhibit EMT more effectively.

It is well known that only having the upregulated expression of TJs/AJs molecules is insufficient for a cell junction, their appropriate locations are the necessary conditions. For example, Claudin-1 was the first discovered and was found to be required for barrier function. Yet it is upregulated in colon cancer, and its expression is unexpectedly promoted by β-catenin/TCF [34]. In IHC of patient biopsies, Dhawan et al. observed a marked upregulation of claudin-1 and displacement from the cell membrane to the cytoplasm and nucleus with tumor progression [35]. In this study, an interesting finding was that although the expression levels of TJs/AJs as well as some of their related molecules, such as ZO-1, claudin 7, E-cadherin, E-catenin, were raised upon N3ICD overexpression, they could not relocate and redistribute back to the cell membrane (data not shown). Differently, the ectopic overexpression of TD+N3ICD could not only upregulate the expression levels of these molecules, but also could contribute to correcting the position of TJs/AJs molecules and facilitate cell-cell adhesion, which is essential for morphogenesis and reversing EMT, in breast cancer MDA-MB-231 cells.

In addition to the core components of TJs/AJs molecules, several multiprotein complexes need to be recruited depending on the cell type and/or developmental stage, which is a prerequisite for the junction between two neighboring cells or a cell with the extracellular matrix, and is needed to maintain apical-basal cell polarity, to form the barriers guarding the intercellular spaces, and to help in establishing communication between neighboring cells. Published studies have shown that the formation of the PAR-3-aPKC-PAR-6 complex and aPKC kinase activity are required for normal apical domain development [36] and for the maintenance of the apicobasal polarity of epithelial cells [37,38]. Accordingly, we paid attention to whether ectopic overexpression of N3ICD or TD+N3ICD impacted those molecules above mentioned. We found that only TD+N3ICD overexpression could activate aPKC and upregulate the expression of PARD3 and PARD6. Of note, their positions tended towards normalization with TD+N3ICD overexpression. It is well known that protein complex containing PAR3/PAR6/aPKC co-localize with Crb complex in the rhabdomere stalk of photoreceptors [39]. Therefore, the mechanism why TD+N3ICD could upregulate the expression of aPKC, PARD3 as well as PARD6, and impel them back to the right positions should be due to the intermediary molecules, Crb complex. In line with our observations, published studies have demonstrated that in mammalian neural development, Notch is positively regulated by the PAR complex proteins Pard3 and aPKC, promoting apical neuroepithelial identity [40,41].

As expected, our study demonstrated that PATJ, a polarity protein, was upregulated and was targeted to the apical region with the overexpression of TD+N3ICD in MDA-MB-231 cells. Tepass, et al. showed that PATJ forms functional complex with Crumbs and PALS1, Crumbs/PALS1/PATJ, in epithelial polarity. We therefore inferred that TD+N3ICD promoted PATJ back to apical region and tight junctions through the interaction between Crumbs and the endogenous full-length Notch3. Our results were partly supported by the previous report that the reduction in PATJ expression by RNAi techniques leads to delayed tight junction formation and causes defects in cell polarization [42].

Owing to the appropriate positions of all above-mentioned molecules, TJs and AJs between neighbor MDA-MB-231/TD+N3ICD cells might be remodeled. The Golgi apparatus areas were correlated inversely with mesenchymal classification and the depletion of GM130 is sufficient to induce E-cadherin downregulation and mesenchymal marker (N-cadherin and vimentin) upregulation, indicative of a loss in cell polarity and epithelial identity [43–45]. Normally, epithelial cell polarization along an apical-basal axis causes Golgi vesicles to coalesce into compact ribbon structures that face the apical surface and direct vesicle trafficking toward apical and basolateral membrane compartments [46]. During EMT, the Golgi is repositioned to direct vesicle trafficking toward the leading edge of the cell and facilitate the formation of promigratory focal adhesions and cytoplasmic protrusions [47]. Accordingly, the locations of *cis*-Golgi marker GM130 were used to verify the transition from mesenchymal to epithelial as well as reconstruction of TJs and AJs in this study. Very typically, the positive signals of GM130 with higher intensity were mainly positioned at the leading edge of MDA-MB-231/pCMV cells, whereas upon TD+N3ICD overexpression, weaker signals of GM130 dispersedly located to the inner side of a membrane.

Unexpectedly, another adhesion molecule, alpha-integrin 6, which connects cells with extracellular matrix molecules of the laminin family, is closely associated with EMT. In this study, we observed a slight change in its expression level with the overexpression or knockdown of Notch3. Interestingly, however, the distribution of alpha-integrin 6 became more uniform encircling the cell-membrane under the condition of TD+N3ICD overexpression. These data indicate that the increased Notch3 signaling pathway activity can reverse EMT or promote mesenchymal-epithelial transition (MET).

Finally, we were also interested in the molecular mechanism of the restored cell adhesion in MDA-MB-231/TD+N3ICD cells. Comparing the difference between the overexpression of N3ICD and TD+N3ICD, we found that TD+N3ICD overexpression could upregulate the expression of endogenous full-length Notch3. We believed that the upregulated endogenous full-length Notch3 may be an important clue. At first, Notch receptor and ligand co-exist within the same cells and neighboring cells and affect each other in a reciprocal manner. The Notch signaling requires direct contact between signal-sending and signal-receiving cells. Therefore, the upregulation of full-length Notch3 provides more chances for the homophilic E-cadherin binding among bilaterians that are necessary for intrinsic homophilic cell–cell adhesion activity [48]. Various experimental results have suggested that lateral interactions may be an important feature of cadherin adhesive functions [49]. And then, we have found the direct interaction between the transmembrane full length Notch3 and E-ca according to the result of Co-IP, which will help disassembled E-ca to relocate to cell membrane. In line with our findings, Kikuchi, et al. reported that Notch2 and the ligand Jagged1 were localized within E-cadherin–positive cells in the marginal layer cells [50]. In addition, TD+N3ICD overexpression could amplify the roles of Notch3 signaling pathway when compared with N3ICD overexpression alone. Once TD+N3ICD was ectopic overexpressed, the endogenous full-length Notch3 was upregulated, which could further activate the expression of downstream target molecules through the canonical Notch pathway. We had noticed that the promoters of many TJs/AJs as well as some of their related molecules (e.g., E-ca, ZO-1, CLDN4, INADL, Crumb) contained an RBP-jκ-binding site, implying that these genes may be the direct targets of the Notch3 signaling pathway.

Collectively, our findings showed that the overexpression of N3ICD combined with the transmembrane domain is sufficient to upregulate various TJs/AJs molecules and apical-basal cell polarity complex and drive them to appropriate positions, and thereby inhibit or reverse EMT by canonical and noncanonical pathways simultaneously. Further studies are needed. For example, we will also test the permeability of breast cancer cell TJs by trans-epithelial resistance measurements (TER) in the conditions of Notch3 overexpression or knockdown.

## Materials and methods

### Cell lines, antibodies and reagents

MDA-MB-231 and MCF7 breast cancer cell lines were purchased from the Committee on Type Culture Collection of the Chinese Academy of Science (Shanghai, China). Cells were routinely grown with DMEM supplemented with 10% fetal bovine serum and 1% penicillin/streptomycin. Cells were passaged every 2–3 days and the medium changed once between splits. Once these cultures reached confluence, the cells were used for various experiments such as qRT-PCR and western blotting. The antibodies used in this study are shown in Table S1. All cell lines had been authenticated and tested. There was no mycoplasma contamination.

### Vectors and transient and stable transfection

The eukaryotic expression plasmids pCLE and pCLE-N3ICD were stored in our laboratory. The Notch3 containing transmembrane domain overexpression mammalian cell vector, pCMV/(TD+N3ICD) (OmicsLink^TM^ Expression Clone, CMV promoter), was constructed by GeneCopoeia, Inc. Notch3 interfering plasmid made using pGPU6/GFP/Neo as backbone was purchased from GeneCopoeia, Inc. The cells were seeded into six-well plate at a density of 1 × 10^5^ cells per well in 2 ml of the appropriate growth medium supplemented with serum. For each transfection, 1 to 2 μg DNA were diluted with 100 μl of serum/protein-free medium, and 2 to 25 μl of Lipofectamine reagent were diluted with 100 μl of serum/protein-free medium. The two solutions were combined with gentle mixing and incubated at room temperature for 15 to 20 min.

The cells were washed once with 2 ml of PBS. For each transfection, 0.8 ml of medium contained 10% FBS but without antibacterial agents was added to each tube containing the lipid:DNA complexes. After gentle mixing, the diluted lipid:DNA solution was overlaid on the washed cells. Medium was replaced at 6 to 8 h following the start of transfection. For transient transfection, depending on cell type and promoter activity, cell extracts were tested for gene activity 24 to 72 h after the start of transfection. For stable transfection, after transfection, cells were allowed to grow and to express the protein for G418 resistance under non-selective conditions for at least 24 h. For the selection of stably expressing cells, cells were cultivated in standard medium with supplements and the appropriate amount of G418, Cells were grown for at least 3 weeks under selection pressure to avoid contamination with non-resistant cells, and G418 concentration could then be reduced after 1–2 weeks.

### Quantitative real-time PCR

A sample of total RNA (2 μg) was reverse-transcribed with 200 U Moloney murine leukemia virus reverse transcriptase (M-MLV, Promega) in the presence of 0.5 mM deoxynucleotide triphosphate, 25 U RNase inhibitor and 10 mM random hexamer primers, in a total volume of 25 µl. The expression levels were normalized to β-actin/GAPDH in this study. PCR primers were designed using Primerbank online (Table S2). Each quantitative real-time PCR was carried out in triplicate, in 25 µl of TB Green Premix Ex Taq II (Tli RNaseH Plus) (Takara). PCR conditions were as follows: 30 s at 95°C for initial denaturation, followed by 40 cycles of 5 s at 95°C and 30 s at 60°C. The temperature was increased from 65°C to 95°C, and the PCR melting curve was made every 0.5°C after the amplification reaction (BIO-RAD, CFX Connect). The mean value of triplicates for each sample was calculated and expressed as the cycle threshold (Ct). For each gene studied, the level of gene expression was then calculated as the ΔCt, the difference between the Ct value of the sample and the Ct value of GAPDH, which was used as an endogenous control. Expression changes in target genes in the experimental group relative to the control group were calculated as follows: fold change = 2^−(ΔCT,Tg-ΔCT,control)^.

### Protein extraction and western blotting

To detect protein expression levels, breast cancer cells were lysed in ice-cold RIPA lysis buffer (Biospes). Homogenates were then centrifuged at 12,000 × g for 20 min at 4°C. Protein content in the clear supernatant was quantified using a bicinchoninic acid kit (Beyotime), and samples were then reduced and stored at −80°C until use. Samples (20–50 μg protein/lane) were separated by SDS-PAGE. Proteins were transferred from the gel onto a PVDF membrane. After protein transfer, PVDF membranes were blocked with a solution containing Tris-buffered saline, 0.1% Tween 20 (TBST) and 5% fat-free milk for 2 h at room temperature. Membranes were then incubated with primary antibodies (See Table S1) overnight at 4°C. Afterwards, blots were treated with horseradish peroxidase-conjugated goat anti-mouse or goat anti-rabbit IgG (1:10,000) in TBST containing 1% (w/v) BSA for 60 min at room temperature while shaking, and immune complexes were detected using an ECL plus detection kit (Thermo). Finally, PVDF membranes were scanned using the Quantity One Imaging system (Bio-Rad). The relative expression of target protein was normalized to that of GAPDH or β-actin. Every experiment was repeated three times.

### Immunofluorescence staining

MDA-MB-231 cells were transfected with pCMV/(TD+N3ICD), pCLE/N3ICD and empty plasmid and seeded on glass coverslips, respectively, for immunofluorescence staining. In detail, the cells were treated with 4% PFA in PBS for 20 min at room temperature, washed by PBS for 3 times, and followed by 10% goat serum in PBS for 30 min at 37ºC without washing, and then incubated with Notch3 and TJs/AJs molecules primary antibodies overnight at 4°C. On the next day, the cells were washed and incubated with fluorescent dye-conjugated secondary antibodies for 1 h at room temperature in the dark (Notch3 for red color, TJs/AJs molecules for green color). Afterwards, they were washed and incubated with mounting medium (containing DAPI) (VECTASHIELD) for 15 min at room temperature and examined with fluorescence microscope. Immunofluorescence staining samples were imaged using a Zeiss ApoTome.2 confocal microscope and analyzed with ZEN software. The quantification of Notch3 and TJs/AJs molecules positive signals were calculated by Image J software, respectively. All values are presented as mean ± SD.

### Transwell migration assay

Cell culture inserts (8 μM pore size; BD) were used to perform migration assays. Transfected cells were serum-starved for 12 h and 2 × 10^4^ MDA-MB-231/pCLE-N3ICD cells, MDA-MB-231/pCMV-(TD+N3ICD) cells and 5 × 10^4^ MCF-7/siN3 cells in serum-free medium were inoculated into the upper chamber, respectively. Complete medium was added to the bottom chamber. Cells were stained with 0.1% crystal violet for migration assays at 48 h (MDA-MB-231/pCLE-N3ICD, MDA-MB-231/pCMV-(TD+N3ICD) and MCF-7/siN3). The assay was performed in triplicate. The number of cells from five fields in each well was determined by three independent investigators. All values are presented as mean ± SD.

### Wound healing assay

Transfected cells were serum-starved for 12 h before a scratch wound was made. Making a scratch wound by 2 mm-wide tip when the cells were grown to 90% confluence. After rinsing with PBS, cells were allowed to migrate in medium containing low serum (1% fetal bovine serum and 1% penicillin/streptomycin), and photographs were taken (×40) at different time points. An average of five random widths along the injury line were measured. All values are presented as mean ± SD.

### Acini formation assay

MDA-MB-231 cells transfected with pCLE, pCLE-N3ICD, pCMV and pCMV-(TD+N3ICD) vectors, respectively. Cells were treated with trypsin, respectively, and first resuspended in complete medium (DMEM medium supplemented with 10% FBS and 1% penicillin/streptomycin). The cells were pelleted through centrifugation at 125 x g for 5 min and then resuspended in complete medium. Count cells concentration by the TC20 automated cell counter (Bio-Rad). Mixing 100μl cell suspension containing 1 × 10^5^ cells and 200μl Matrigel matrix (10 mg/ml) (Corning), which was kept on ice. Using pre-chilled tips to seed the mixture into 24-wells plate. Incubate the plate at 37°C for 30 to 45 min. Gently add 500μl complete medium to each well. Keep culture for 8 to 10 days and change complete medium every 2 days. For further observation of malignant acini development along a time course.

### Co-immunoprecipitation assays

Total proteins from MDA-MB-231/pCMV-(TD+N3ICD) and MDA-MB-231/pCLE-N3ICD, were extracted by IP Lysis (Pierce Classic IP Kit, Thermo). Pre-clear lysate using the Control Agarose Resin. Combine 2 µg Notch3 antibody or Rabbit IgG isotype control antibody with the pre-cleared cell lysate, and then incubate overnight at 4°C. Add the antibody/lysate sample to Protein A/G Plus Agarose and incubate column with gentle end-over-end mixing or shaking for 1 h. Wash the resin three times with 200μl Wash Buffer and centrifuge (1000 × g for 1 min) after each wash. Add the 2X reducing sample buffer, and incubate at 100ºC for 5–10 min. Allow the sample to cool to room temperature before applying to the SDS-PAGE gel. The binding proteins, such as E-ca and Notch3, were probed via western blot.

### Statistical analysis

Statistical analysis was performed using SPSS 18.0 software. Results were presented as mean ± SD. Statistically significant differences were evaluated using Student’s t-test. p < 0.05 was considered significant.
