# Supplementary material for: N3ICD with the transmembrane domain can effectively inhibit EMT by correcting the position of tight/adherens junctions
Source: Cell Adh Migr. 2019 May 27;13(1):203–18. doi: 10.1080/19336918.2019.1619958 (PMC6550553; doi:10.1080/19336918.2019.1619958)
Supplement: Supplemental Material [file kcam-13-01-1619958-s0001.zip › Table 1 antibodies.docx]

**Table 1. Antibodies used in this study**

| Antibody | Cat: # | Company | Con. | Species | |  |
| --- | --- | --- | --- | --- | --- | --- |
| Integrin alpha 6 | ab75737 | Abcam | 1:200 | Rabbit | | IF |
| Claudin 7 | ab27487 | Abcam | 1:50 | Rabbit | | IF |
| Claudin 7 | ab27487 | Abcam | 1:2000 | Rabbit | | WB |
| E-Cadherin | ab15148 | Abcam | 1:100 | Rabbit | | IF |
| E-Cadherin | ab15148 | Abcam | 1:2000 | Rabbit | | WB |
| α-E-Catenin | #3236 | CST | 1:200 | Rabbit | | IF |
| α-E-Catenin | #3236 | CST | 1:2000 | Rabbit | | WB |
| GM130 | PA1-077 | Thermo Fisher | 1:100 | Rabbit | | IF |
| LLGL2 | ab55423 | Abcam | 1:100 | Mouse | | IF |
| LLGL2 | ab55423 | Abcam | 1:2000 | Mouse | | WB |
| NOTCH3(M02) clone 2E2 | H00004854-M02 | Abnova | 1:100 | Mouse | | IF |
| NOTCH3 | #2889 | CST | 1:100 | Rabbit | | IF |
| NOTCH3 | #2889 | CST | 1:1000 | Rabbit | | WB |
| NOTCH3 | #2889 | CST | 1:50 | Rabbit | | CO-IP |
| PARD3 | ab64840 | Abcam | 1:500 | Rabbit | | IF |
| PARD6B | PA5-30854 | Thermo Fisher | 1:100 | Rabbit | | IF |
| PARD6B | PA5-30854 | Thermo Fisher | 1:1000 | Rabbit | | WB |
| PATJ | bs-12142R | Bioss | 1:500 | Rabbit | | IF |
| PATJ | bs-12142R | Bioss | 1:1000 | Rabbit | | WB |
| ZO-1 | ab59720 | Abcam | 1:100 | Rabbit | | IF |
| ZO-1 | ab59720 | Abcam | 1:2000 | Rabbit | | WB |
| CRB3 | sc-292449 | Santa Cruz | 1:500 | Rabbit | | WB |
| RBPJK | ab25949 | Abcam | 1:2000 | Rabbit | | WB |
| Vimentin | #5741 | CST | 1:2000 | Rabbit | | WB |
| aPKC | MBS624797 | Mybiosource | 1:1000 | Rabbit | | WB |
| His-Tag | 66005-1-Ig | Proteintech | 1:5000 | Mouse | | WB |
| β-actin | 100162-RP02 | Sino biological | 3:2000 | Rabbit | | WB |
| GAPDH | TA-08 | ZSGB-BIO | 1:3000 | Mouse | | WB |
|  |  |  |  |  | |  |
| Rabbit IgG Isotype Control | #3900 | CST | 1ug/mg protein | Rabbit | | CO-IP |
|  |  |  |  |  | |  |
| Secondary antibodies | | | | | | |
| Goat Anti-Rabbit IgG (H+L) | ZB-2301 | ZSGB-BIO | 1:3000 | | Goat | WB |
| Goat Anti- Mouse IgG (H+L) | ZB-2305 | ZSGB-BIO | 1:3000 | | Goat | WB |
| Goat Anti-Rabbit IgG (H+L) | orb229657 | Biorbyt | 1:5000 | | Goat | WB |
| Peroxidase-Conjugated Goat anti-Mouse IgG（H+L） | ZB-2305 | ZSGB-BIO | 1:5000 | | Goat | WB |
| Goat anti-Mouse IgG (H+L) Cross-Adsorbed Secondary Antibody, Alexa Fluor 594 | R37121 | Thermo Fisher | 2 drops/mL | | Goat | IF |
| Goat anti-Rabbit IgG (H+L) Cross-Adsorbed Secondary Antibody, Alexa Fluor 594 | R37117 | Thermo Fisher | 2 drops/mL | | Goat | IF |
| Goat anti-Rabbit IgG (H+L) Cross-Adsorbed Secondary Antibody, Alexa Fluor 488 | R37116 | Thermo Fisher | 2 drops/mL | | Goat | IF |
| Goat anti-Mouse IgG (H+L) Cross-Adsorbed Secondary Antibody, Alexa Fluor 488 | R37120 | Thermo Fisher | 2 drops/mL | | Goat | IF |
