# Supplementary material for: N3ICD with the transmembrane domain can effectively inhibit EMT by correcting the position of tight/adherens junctions
Source: Cell Adh Migr. 2019 May 27;13(1):203–18. doi: 10.1080/19336918.2019.1619958 (PMC6550553; doi:10.1080/19336918.2019.1619958)
Supplement: Supplemental Material [file kcam-13-01-1619958-s0001.zip › Table2 primers.docx]

**Table 2. Primers used in this study**

| Gene | Forward primer | Reverse primer | purpose |
| --- | --- | --- | --- |
| NOTCH3 | AGCTTGGGAAATCAGCCTTAC | TGCTATCCTGCATGTCCTTATTG | RT-PCR Validation |
| GAPDH | AAGCTCATTTCCTGGTATGACAACG | TCTTCCTCTTGTGCTCTTGCTGG | RT-PCR Validation |
| E-cadherin | ATGTGTTTGTGTGCGACTGC | ACATTGTCCCGGGTGTCATC | RT-PCR Validation |
| CLDN3 | AACACCATTATCCGGGACTTCT | GCGGAGTAGACGACCTTGG | RT-PCR Validation |
| CLDN4 | TGGGGCTACAGGTAATGGG | GGTCTGCGAGGTGACAATGTT | RT-PCR Validation |
| CLDN7 | AGCTGCAAAATGTACGACTCG | GGAGACCACCATTAGGGCTC | RT-PCR Validation |
| CLDN12 | TGTACGACACTACTTGGTACTCA | TTGCACATTCCAATCAGGCAG | RT-PCR Validation |
| TJP1 | ACCAGTAAGTCGTCCTGATCC | TCGGCCAAATCTTCTCACTCC | RT-PCR Validation |
| TJP2 | GGGAAGGTCGCTGCTATTGT | CTCTCGCTGTAGCCACTCC | RT-PCR Validation |
| YBX3 | ACCGGCGTCCCTACAATTAC | GGTTCTCAGTTGGTGCTTCAC | RT-PCR Validation |
| PARD6A | AGCATCGTCGAGGTGAAGAG | GTATAGCCAAGTAGCACGTCC | RT-PCR Validation |
| PARD3 | CAGGTGCATCGCTTGGAAC | GCTGAGACATTGTTGGTGCC | RT-PCR Validation |
| PRKCA | GTCCACAAGAGGTGCCATGAA | AAGGTGGGGCTTCCGTAAGT | RT-PCR Validation |
| SCRIB | CCTCTGTCAAGGGAGTGTCG | CCCGAGAGATGAATATGCCCTC | RT-PCR Validation |
| DLG1 | TACTCCCCAGTTTCTAAAGCAGT | CAGGTCCTCCGGCTAAGATAA | RT-PCR Validation |
| LLGL2 | CGGGACCTGTTCCAGTTTAAC | CGTCACAGCGTTGTTCTCC | RT-PCR Validation |
| Crb3A | CCCTCTCCTTTCTTTCAGTTCTC | CAGCAATGAAGGGAGACAGAT | RT-PCR Validation |
| INADL | ACTCAGTCATTCAACAGATGGC | CCCTGTCTGCTACACTCCCT | RT-PCR Validation |
| MPP5 | TTACTGGCCCACGATAAGGTT | CACGAGCCTTTTCTATACGAACT | RT-PCR Validation |
| Vimentin | GACGCCATCAACACCGAGTT | CTTTGTCGTTGGTTAGCTGGT | RT-PCR Validation |
