# Supplementary material for: N3ICD with the transmembrane domain can effectively inhibit EMT by correcting the position of tight/adherens junctions
Source: Cell Adh Migr. 2019 May 27;13(1):203–18. doi: 10.1080/19336918.2019.1619958 (PMC6550553; doi:10.1080/19336918.2019.1619958)
Supplement: Supplemental Material [file kcam-13-01-1619958-s0002.zip › supplementary figure legends(junyu 20190322).docx]

**Supplementary Figure legends**

**Figure S1: Double-label immunofluorescence staining with ZO-1 and Notch3 antibodies and z-stack analysis with ZEISS Image. M2 (ApoTome.2) microscopy. Z-stack images collected at 1.11μm sections were presented. The result revealed that the expression of ZO-1 (green color) does overlap with Notch3 staining (red color) on the membrane in MDA-MB-231/pCMV-(TD+N3ICD) cells (Figure S1B) when compared with that in MDA-MB-231/pCMV cells (Figure S1A). 40×.**

**Figure S2: Double-label immunofluorescence staining with E-ca and Notch3 antibodies and z-stack analysis with ZEISS Image. M2 (ApoTome.2) microscopy. Z-stack images collected at 1.11μm sections were presented. The result revealed that the expression of E-ca (green color) does overlap with Notch3 staining (red color) on the membrane in MDA-MB-231/pCMV-(TD+N3ICD) cells (Figure S2B) when compared with that in MDA-MB-231/pCMV cells (Figure S2A). 40×.**

**Figure S3: Double-label immunofluorescence staining with GM130 and Notch3 antibodies and z-stack analysis with ZEISS Image. M2 (ApoTome.2) microscopy. Z-stack images collected at 1.11μm sections were presented. The result revealed that, unlike the pattern of GM130 presented in MDA-MB-231/pCMV cells which are compressed in the cells (Figure S3A), the expression of GM130 (green color) in MDA-MB-231/pCMV-(TD+ N3ICD) cells more evenly distribute around the membrane (Figure S3B), 40×.**
