# Supplementary figures and images for: N3ICD with the transmembrane domain can effectively inhibit EMT by correcting the position of tight/adherens junctions
Source: Cell Adh Migr. 2019 May 27;13(1):203–18. doi: 10.1080/19336918.2019.1619958 (PMC6550553; doi:10.1080/19336918.2019.1619958)

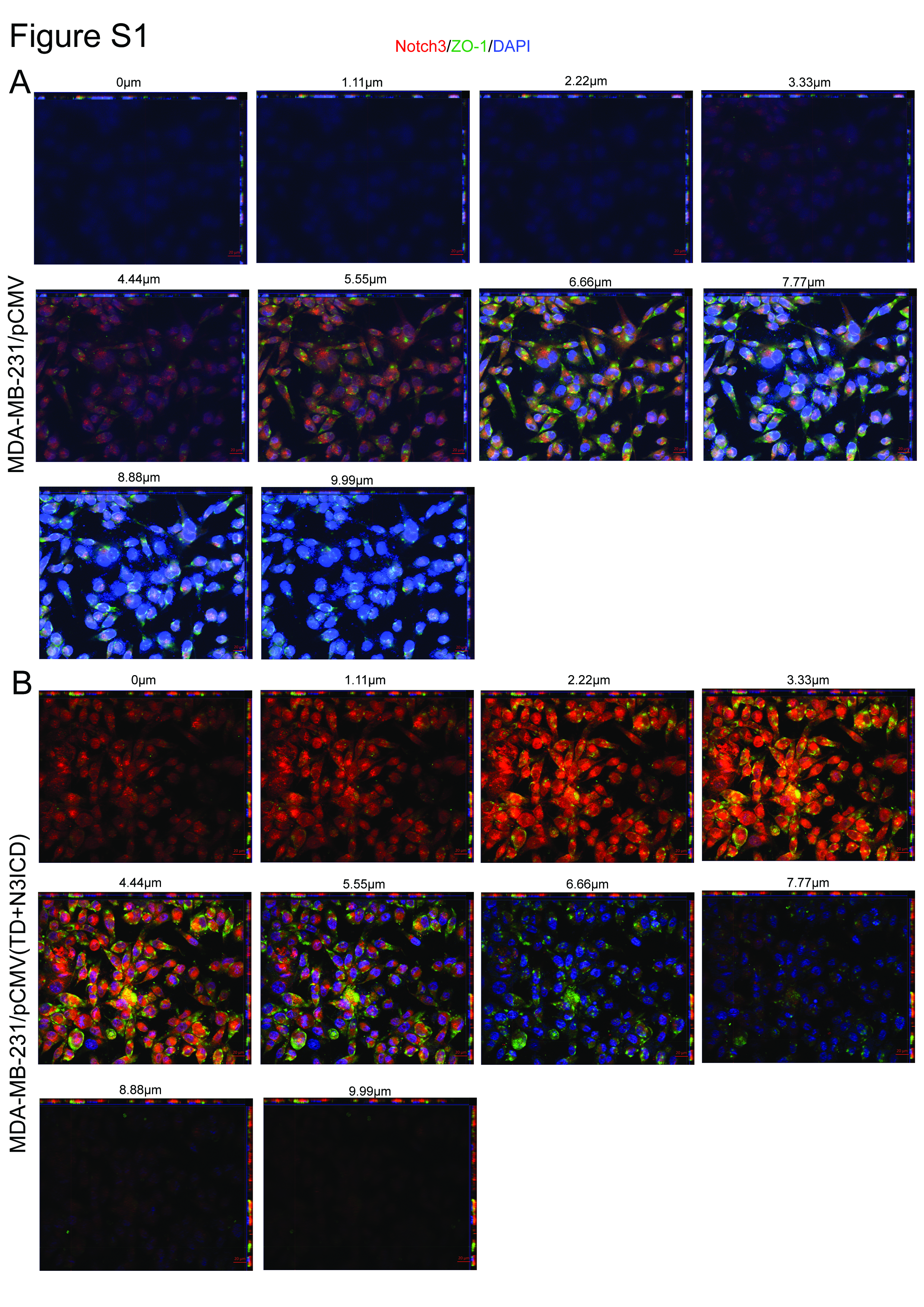

Supplement: Supplemental Material [file kcam-13-01-1619958-s0002.zip › Fig S1.tif]

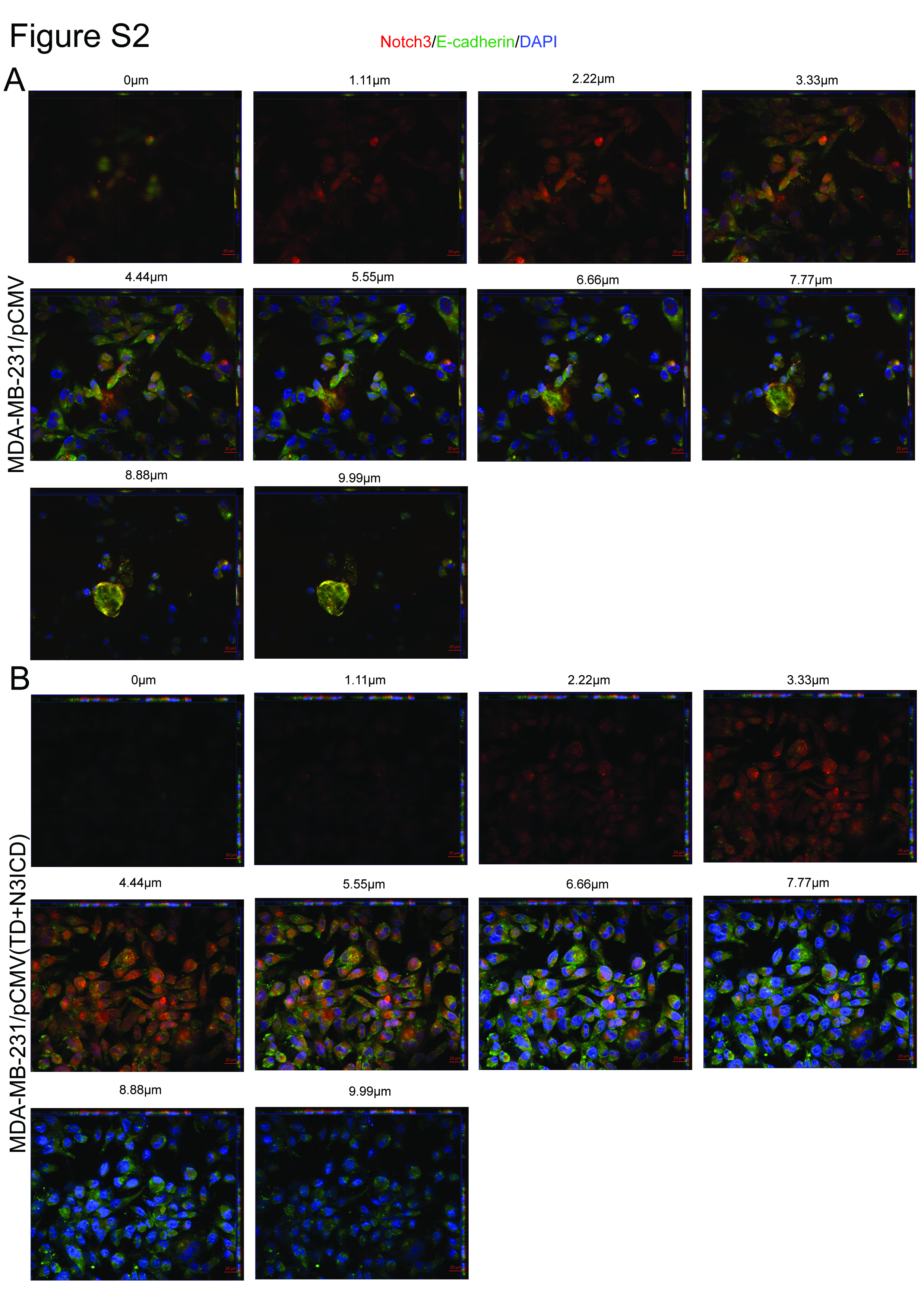

Supplement: Supplemental Material [file kcam-13-01-1619958-s0002.zip › Fig S2.tif]

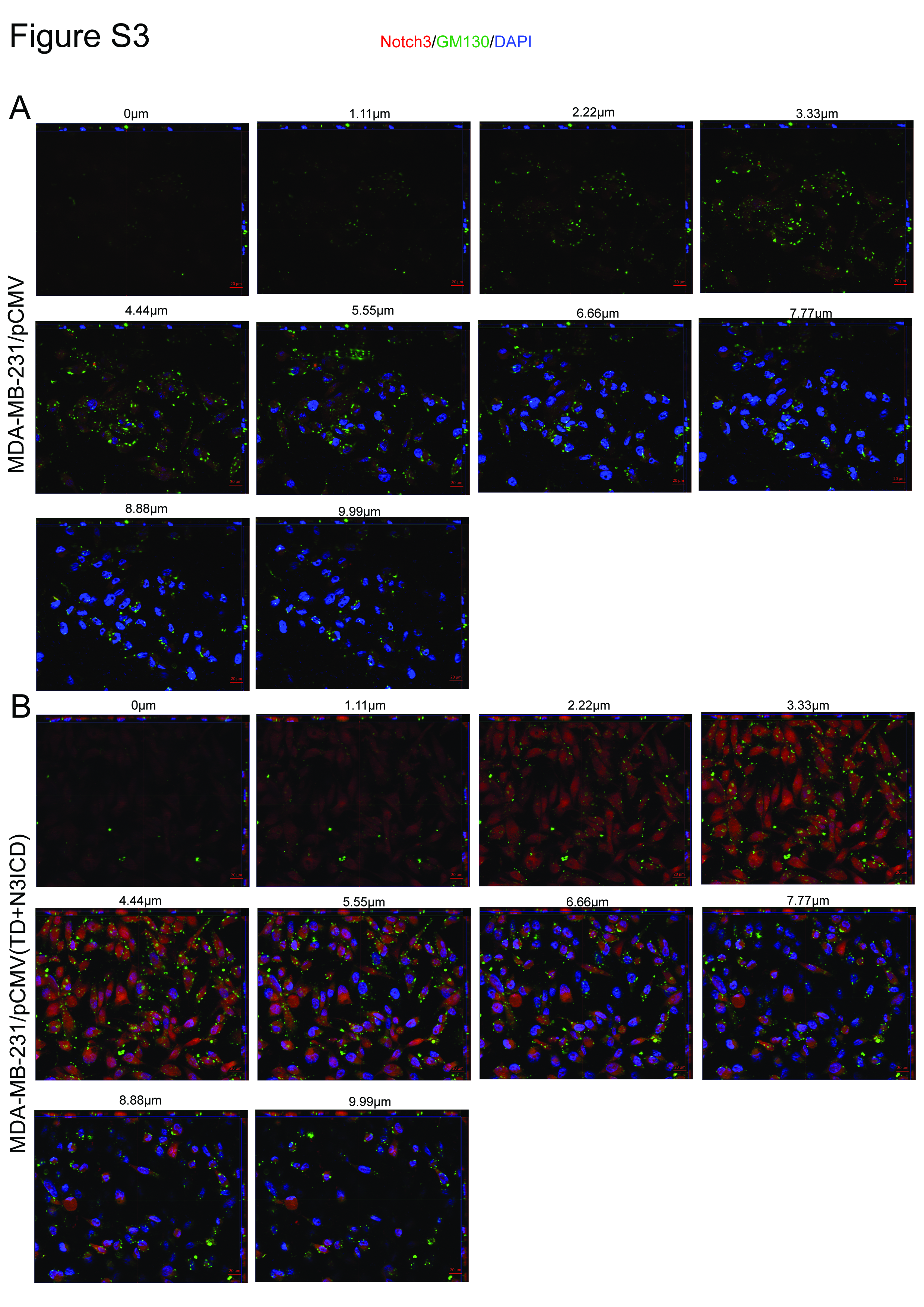

Supplement: Supplemental Material [file kcam-13-01-1619958-s0002.zip › Fig S3.tif]
